# Outpatient teaching in specialist practices – a qualitative study with doctors about attitudes, influencing factors and specialist features

**DOI:** 10.3205/zma001575

**Published:** 2022-11-15

**Authors:** Sven Schulz, Miriam Hesse, Anni Matthes, Inga Petruschke, Jutta Bleidorn

**Affiliations:** 1 Jena University Hospital, Institute of General Practice and Family Medicine, Jena, Germany

**Keywords:** medical training, outpatient teaching, specialist, teaching motivation, teaching practice

## Abstract

**Aim:**

The aim is to record existing attitudes, influencing factors and specialised requirements with regard to the training of medical students in specialist outpatient care with doctors working in outpatient care.

**Method:**

Between September 2020 and May 2021, individual interviews with 15 specialists employed in outpatient care were held as part of this qualitative study. The recorded interviews were evaluated structurally in accordance with the Kuchartz method.

**Results:**

Enhanced outpatient training for medical students in specialist teaching practices was considered as important by all participants. Among other things, motivational factors were pleasure in teaching, a feeling of duty, the desire to pass something on, the need to convey one’s own ideas and to generate future talent for the field. A lack of time, greater organisational effort and legal concerns were stated as hurdles. Reducing organisational effort for teaching practices, appreciation of their teaching activity and financial considerations were in particular given as significant incentives for participating. The attitude of participants towards financial remuneration was heterogeneous. Different specialist features and requirements for student training were mapped out.

**Conclusion:**

This study provides, for the first time, findings about teaching in specialist outpatient establishments. They point to a great degree of willingness of specialist doctors to undertake teaching and provide starting points for creating concepts about producing teaching practices in specialist fields. Further quantitative investigation is required to substantiate the findings before us.

## Zusammenfassung

**Zielsetzung:**

Ziel ist es, vorhandene Einstellungen, Einflussfaktoren und fachbezogene Voraussetzungen hinsichtlich der Ausbildung Medizinstudierender in der fachspezialistischen ambulanten Versorgung bei ambulant tätigen Ärzt*innen zu erfassen.

**Methodik:**

In dieser qualitativen Studie wurden im Zeitraum September 2020 – Mai 2021 leitfadengestützte Einzelinterviews mit 15 ambulant tätigen Fachspezialist*innen durchgeführt. Die per Audio aufgenommenen Interviews wurden inhaltlich strukturierend nach Kuckartz ausgewertet.

**Ergebnisse:**

Eine verstärkte ambulante Ausbildung Medizinstudierender in fachspezialistischen Lehrpraxen wurde von allen Teilnehmer*innen als wichtig erachtet. Motivatoren waren u.a. die Freude an der Lehrtätigkeit, ein Verpflichtungsgefühl, der Wunsch etwas weiterzugeben, das Bedürfnis eigene Ideale zu vermitteln und die Nachwuchsgenerierung für das Fach. Als Hürden wurden fehlende Zeit, hoher organisatorischer Aufwand und rechtliche Bedenken geäußert. Als wesentliche Anreize zur Beteiligung an der studentischen Lehre wurden insbesondere die organisatorische Aufwandreduktion für die Lehrpraxen, Wertschätzung ihrer Lehrtätigkeit und finanzielle Vergütung angesprochen. Die Haltung der Teilnehmer*innen zur finanziellen Vergütung war heterogen. Unterschiedliche, fachspezifische Besonderheiten und Voraussetzungen für die studentische Ausbildung wurden herausgearbeitet.

**Schlussfolgerung:**

Mit dieser Studie liegen erstmals Erkenntnisse zur Lehre in fachspezialistischen ambulanten Praxen vor. Sie weisen auf eine hohe Bereitschaft der Ärzt*innen zur Durchführung der Lehre hin und geben Ansatzpunkte für Konzepte zur Lehrpraxengewinnung in den fachspezialistischen Fächern. Es sind weiterführende quantitative Untersuchungen erforderlich, um die vorliegenden Ergebnisse zu untermauern.

## Introduction

The medical study 2020 master plan stipulates focusing on enhanced integration of outpatient practices in medical training [1]. The new regulations for licensing doctors will also result in containing greater consolidation of teaching in the field of outpatient care. In particular, in the practice year (PJ) divided into quarters, a practice quarter in outpatient care should be completed, either in general practice or another point-of-care specialism. While outpatient teaching has been established since 2006 with placements over several weeks in medical studies in the general practice department, the inclusion of specialist clinics is a new feature in the training of medical students on a federal level. The first to approach systematically including outpatient institutions or, as may apply, specialist teaching surgeries in the training of medical students are the Heidelberger Modellstudiengang MaReCuM [2][2], Jenaer Neigungsorientierte Medizinstudium JENOS [3] and the “Praxis-Track” of Goethe Universität Frankfurt am Main [4]. While university outpatient clinics are primarily involved in MaReCuM as outpatient teaching institutions, approximately 70 specialist teaching practices, primarily from Thuringia, take part in JENOS. 

There is a lack of more recent literature, such as Franco et al. in a narrative review on barriers for outpatient training [5] and more often than not refers to literature about GPs [6], [7].

In Germany, we are unaware of any studies of specialist outpatient practices’ views on student teaching, what motivation they have in this respect and what they are influenced by. 

The available German-language literature on the motivation and attitude of GPs points to a high degree of motivation for involvement in student teaching [8], [9]. Specific reasons for participating in student teaching are, among other things, the passing on of knowledge and experience, the development of young talent, and the enrichment of day-to-day surgery life [10], [11]. Potential barriers are concerns about extra time spent, stress and increased organisational effort [8], [10], [11]. 

Due to the differences between general and specialist practices (clinical pictures, practice structures, working methods), these findings are only transferable to a certain extent. 

## Aim of the work

The aim was to record existing attitudes, influencing factors and specialised requirements with regard to the training of medical students in specialist outpatient care with doctors working in outpatient care. 

## Methods

### Study design

We undertook a cross-sectional study with structured individual interviews in the period September 2020 to May 2021 in Thuringia. 

#### Participants and recruitment

Doctors employed in outpatient care in Thuringia were recruited for the study. Specialist doctors for general medicine and those specialising in internal medicine in general practice were excluded as there is already extensive data and awareness available regarding teaching motivation and the structural engagement of general medical practices in university teaching. A selectively chosen random sample of 15 to 20 interview participants was sought to achieve a broad spectrum of ages, a balanced gender distribution and heterogeneity of disciplines. Participants were initially recruited by advertising the study at a teaching practice meeting of Jena University Hospital. Doctors were also contacted by phone or email in a targeted manner. Participation in the study was voluntary and could be retracted at any time. There was no allowance for expenses. Of 45 doctors contacted, 15 took part in the study.

#### Interview guidelines and data collection

The semi-structured interview guidelines were developed, piloted and finalised in the period June to August 2020. In particular, it included the topics attitudes towards outpatient teaching, one's own motivation, hurdles and incentives as well as specialism-specific features. Interviews were either held at the participant’s place of activity or via an online meeting, and saved as an audio file. Before the commencement of the interview, an accompanying questionnaire about socio-demographic data was completed by the participants. The interviews lasted between 22 and 50 minutes.

#### Data analysis

The audio files were transcribed with F4 software in accordance with established rules. Finally, the interviews were pseudonymised. The structural content was analysed in accordance with the Kuckarts method using MAXQDA version 2018 software. A medical student (MH), a psychologist (AM) and a specialist for general practice (SvS) took part in the evaluation. By means of inductive and deductive category formation, MH created an initial system of categories, based on the first two interviews. This was edited and completed by AM and SvS by consensual agreement. The remaining interviews were coded by MH. Inconclusive parts of text were discussed in the working group.

The socio-demographic questionnaires were evaluated statistically and descriptively with the help of Excel software.

#### Ethics

The ethics committee of Jena University Hospital checked and approved the study project (quality mark 2021-2226-Bef).

## Results

### Socio-demographics

A total of 15 doctors from the following specialisms were able to be surveyed: Surgery/orthopaedics (n=4), internal medicine (n=2), neurology/psychiatry (n=2), anaesthetics, ophthalmology, dermatology, gynaecology/midwifery, ENT, paediatrics and radiology/radiotherapy/nuclear medicine (n=1 each). 10 of the doctors took part in the JENOS project. The socio-demographic features are set out in table 1 (Tab. 1).

#### Attitude to outpatient teaching in specialist practices

Increased outpatient training in specialist training practices for medical students was overall endorsed and considered as important by all interviews. This positive attitude was borne out both among the participants already involved in medical training as part of JENOS and also participants not already involved in teaching. Three different weightings could be differentiated. One part observes this development rather neutrally, but with curiosity and expresses that it is *“positively surprised”* (I2, A49) that this shift should take place, and describes it as* “important and useful”* (I8, A25). Another group welcomes the development, but appears to be rather sceptical with regard to implementation.* “This is a real troubled story. Therefore --- I still..., I had no idea. How it can actually work”* (I1, A105). From their point of view it is a *“major project”* (I1, A126), which *“can only be implemented under appropriate conditions”*. The third group of interviewees expressed itself to be very clearly supportive of the development:* “...more outpatient teaching coming into play is absolutely necessary”*. (I7, A3). One person even regarded it as* “absolutely necessary”* as* “one would not succeed without the other”* (I11, A5), meaning with this the clinical and outpatient sector. Some of those surveyed were also of the opinion that it was a development that should have been envisaged long ago and would welcome implementation as soon and expediently as possible.

Their own experience of multiple shifting of care to the outpatient sector and the increasing dovetailing of the inpatient and outpatient sectors was cited as justification for these fundamentally positive approaches to outpatient student teaching. Furthermore, this goes with the fact that a recruitment problem in the outpatient sector acting to counteract it will occur in the future. For this reason, giving students the opportunity during study of becoming acquainted with the specific outpatient way of working in addition to the potential of the branch is important. They should therefore also gain practical experience in the outpatient branch, as many of those surveyed would have themselves wanted an earlier insight. This also requires, in part, revising *“completely incorrect ideas on how outpatient medical science works”* (I2, A91).

#### Motivation, hurdles and incentives

The influencing factors and feasibility of student teaching in one’s own specialist practices can be classed into motivation, hurdles and incentives. The pleasure in teaching, a sense of commitment, the desire to pass something on and the need to convey one's own ideals are stated as intrinsic motivators. There is also the wish to do something better than experienced when studying oneself. Further motivators were bringing up new blood for the field, the search for successors in their own practice as well as the desired enrichment in the sense of a win-win situation (I6, A59 & I9, A47) that could be brought about by students in their own practice. 

In particular, the lack of time and one's own time management or, as may apply, the quick pace in running an outpatient practice were cited as hurdles. Teaching costs time and not only represents an additional burden, but to a certain extent also a “disruptive factor”, which could lead to financial losses by slowing down the running of the practice. On the other hand, participants stated that the financial remuneration for teaching activity played a somewhat minor role. Added to this are worries about greater organisational effort, additional duties and stricter requirements. Legal concerns were also expressed. Some of those surveyed also asked the question their patients and colleagues or employees accepting students in the practice. Personal factors such as insecurity about individual suitability or fear of contact were also brought up. With a view to other colleagues, participants suspected that general resignation and frustration with regard to their activity in the field of outpatient care may represent a hurdle.

In particular, the areas of expenditure reduction, financial compensation and appreciation of teaching activity were cited as significant incentives or, as may apply, beneficial factors for partaking in student teaching in the practice. Reducing expenditure for the teaching practice included sufficient administration and organisation of teaching by the university, including student selection and allocation, clarification, insurance and liability questions, the establishment of fixed and easily contactable points of contact and the easy invoicing of teaching activity. The desire for clear structural specifications for teaching content was stated, and at the same time there should be autonomy when implementing the teaching. Continual training events to the teaching or teaching practice meetings were given as important opportunities for information and discussion. Nevertheless, they should be as concise as possible and take place as close to the practice as *“motivation decreases with every kilometre”* (I7, A31). In addition, frequency, allowances and physical well-being during the event were cited as relevant aspects. 

The participants’ attitude to financial remuneration for the teaching was varied. Some found a financial allowance as appropriate, and in some instances as absolutely necessary. One significant argument was the loss of earnings due to time spent on student support. One participant stated that €500 for a three-week placement was sufficient, while another regarded €100 per day as appropriate. A proportion of those surveyed regarded financial compensation with scepticism. Teaching practices should not be dependent on payment for teaching and this does not represent any additional motivational incentive. 

Instead, it should be appreciated in a different way. Recognition in the form of CME points could be one potential incentive. In this case, close cooperation is required with the State Medical Council and the Association of Statutory Health Insurance Physicians, as well as recognition of their commitment by these institutions. The official designation for the teaching practice including the certificate was also named as the form of the appreciation and recognition. 

#### Specialised features

In general, it became clear that a different range of syndromes and working structures is present in the specialist disciplines. These aspects may vary within the teaching practice of a specialist discipline. In addition to this, individual specific features for the various specialist areas were cited (see table 2 (Tab. 2)).

## Discussion

The aim of this study was to obtain insights for undertaking outpatient teaching in specialised practices. In particular for the German-speaking area, systematic attitudes and influencing factors among doctors employed in outpatient care and specialised features were gathered.

### Attitudes

Overall, a consistently positive attitude of the participants with regard to the predicted development of undertaking medical training in specialist outpatient practices is demonstrated. This favourable attitude is justified with a clear necessity for reinforced outpatient (specialist) training – by the increasing shift of medical care to the outpatient sector and a simultaneous increasing problem of new talent. The problem with succession may refer to both general care in the respective specialist area and the specific succession in one's own practice. Specialist area with a high proportion of independent health insurance scheme doctors in Thuringia are, in particular, ophthalmology and neurology/psychiatry [12].

The interview partners' estimates correspond accordingly to the aspirations of the 2020 medical study master plan. Even if all participants endorse the development in principle, the estimates about the feasibility of increased specialist outpatient training vary from sceptical to optimistic. In this case, in future studies, whether this estimate refers to one's specific own practice or a comprehensive implementation should be differentiated.

Furthermore, what these evaluations are based on is left open by this study. In this way, missing or one's own previous experiences could play a role.

#### Motivations

The motivations for taking part in outpatient teaching reveal a diverse range and correspond partly to findings from general practice studies. As a result, conveying practical skills and knowledge is known to be a significant motivating factor for taking part in teaching [9]. Qualitative research with Australian general practitioners pointed to the updating of one’s own clinical knowledge as the most frequently stated motivator [7]. Additional motives for general practitioners were personal development, the pleasure in teaching, the requirement to convey one's own specialism as a career option, and a feeling of responsibility towards the profession and society [7], [13]. These positive aspects may be used for acquiring new specialist teaching practices and increasing their willingness to participate in student teaching.

#### Hurdles and incentives

A lack of time was cited as the most significant hurdle for undertaking outpatient teaching. It is also depicted this way among GPs [7], [8]. Interestingly, uncertainty with regard to this study’s participant’s own suitability was cited. Here, ambivalence between the will of the participants and the lack of security appears to be represented. Nevertheless, this may be countered by suitable continual training measures by the faculties. Some hurdles, for example the great amount of work required, are represented reciprocally among the stated incentives in the sense of a reduction in organisational expenditure and structures and points of contact being established by the faculties. The reduction in expenditure also refers to the intensity of teaching. This includes the number of contacts between teachers and students, the length of contacts and their content [14]. When GPs from Saxony were surveyed about the amount of time required, an average period of 6.9 hours per month made available by doctors for teaching was stated [9]. Continual training events for teachers have not been depicted in the literature as incentives. In the research by Klement et al., 66% of those surveyed agreed that teaching doctors should take part in continual teaching practice training [9].

Remuneration is depicted as a more important, yet more valued incentive. As such, whether remuneration is regarded as compensation or as a fee appears to be of significance. In the investigation by Deutsch et al. with GPs from Saxony, compensation for productivity reduced by student teaching was regarded as important. Nevertheless, in this case there was a proportion of GPs who would undertake teaching without remuneration [8], as was also the case in the survey undertaken by us. In Klement's investigation, a fee was important for 50% of participants [9]. In further investigations, the view of specialists on the type and amount of remuneration should be quantitatively recorded in order to make sufficient remuneration available in consideration of the universities’ resources. 

Over and above remuneration, intangible recognition represents a significant incentive for partaking in teaching. The appreciation perceived of their commitment being of greater significance for the doctors surveyed by us was clear. Collaboration with external university institutions inspired by interview partners is to be highlighted. Incentives, for example the award of CME points, for which medical associations are responsible, could be created in this case in consideration of the faculties' statutory tasks for student training.

#### Specialised features 

Over and above the general heterogeneity of departments with regard to clinical pictures, working structures and equipment, specialised requirements and challenges for successful teachers in individual specialist disciplines stood out. Recording these in connection with doctors working in outpatient care and developing specialist teaching content and methods on this basis is essential for the further development of student training in an outpatient setting. 

#### Limitations

When interpreting the findings of this study, it should be borne in mind that the planned selection of participants by gender and subject field could not be implemented in full. As such, gender distribution was not balanced. In addition, the heterogeneity of specialist fields was restricted and not all specialist fields are represented in the research. In part, specialist fields were only presented by one person. Limitation of participant numbers and local restriction to Thuringia doctors was conditioned by limited resources. A further limitation consists in the selection of doctors surveyed in the study, of which a major proportion is already actively involved in project JENOS into outpatient teaching. As such, the transferability of the findings is restricted in particular for non-considered specialist fields, regions differing greatly from Thuringia and doctors hardly coming into contact with teaching to date. Further, as may apply, quantitative studies are required to reinforce the significance of the findings. The findings of this qualitative study serve as a basis for more in-depth studies and offer an initial starting point for implementing outpatient teaching in specialist practices in a targeted and needs-based manner.

## Conclusion

This study provides, for the first time, findings about teaching in specialist outpatient establishments. They point to a greater willingness of specialist doctors to undertake teaching, allow a positive outlook for feasibility and provide starting points for creating concepts about producing teaching practices in specialist fields. Requirements specific to specialist areas were clear, which are worth considering when further developing teaching in an outpatient setting. In addition, further or, as may apply, specialist quantitative investigations are nevertheless required to underpin and substantiate the findings before us. 

## Competing interests

The authors declare that they have no competing interests. 

## Figures and Tables

**Table 1:**
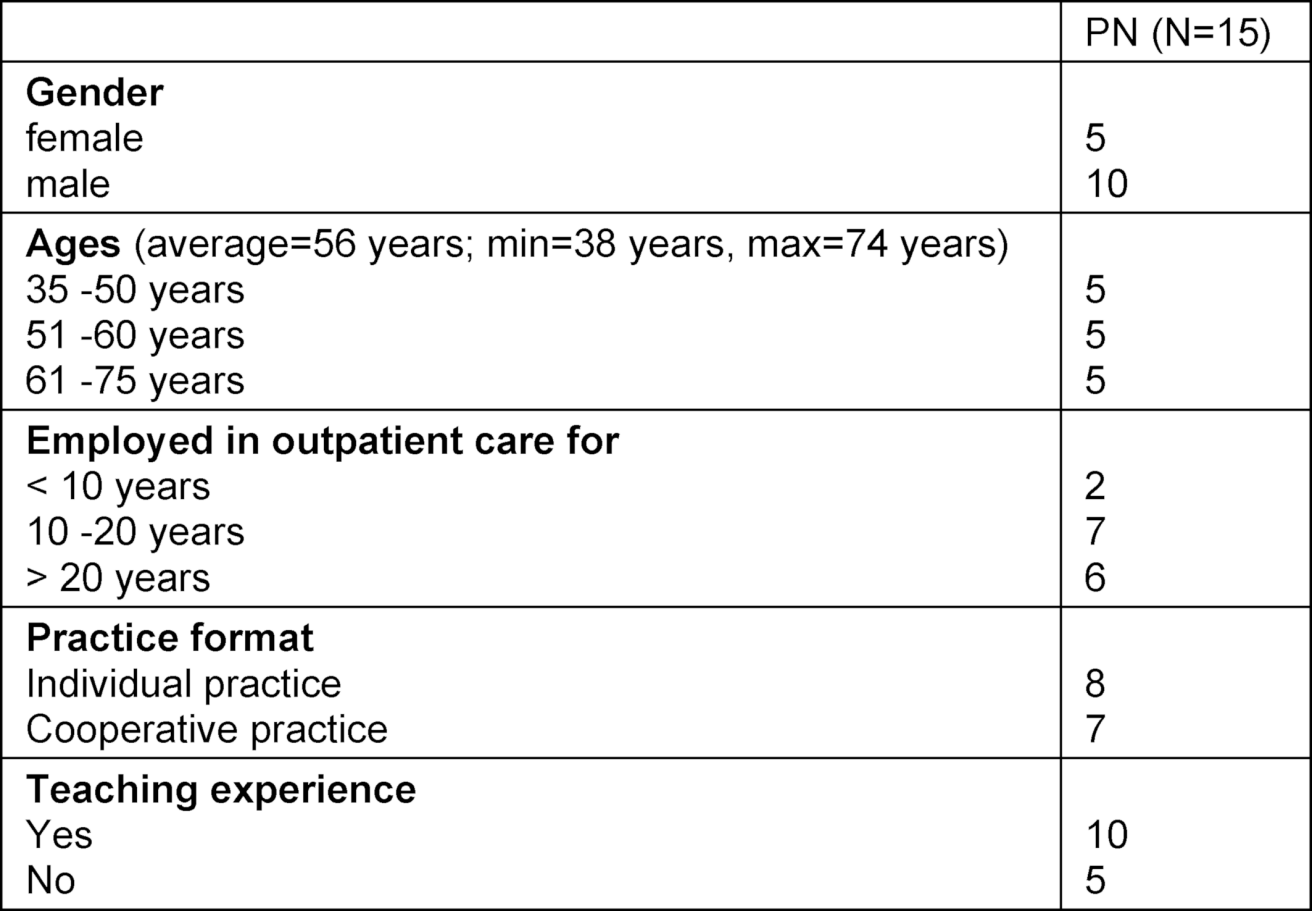
Socio-demographic characteristics of the participants

**Table 2:**
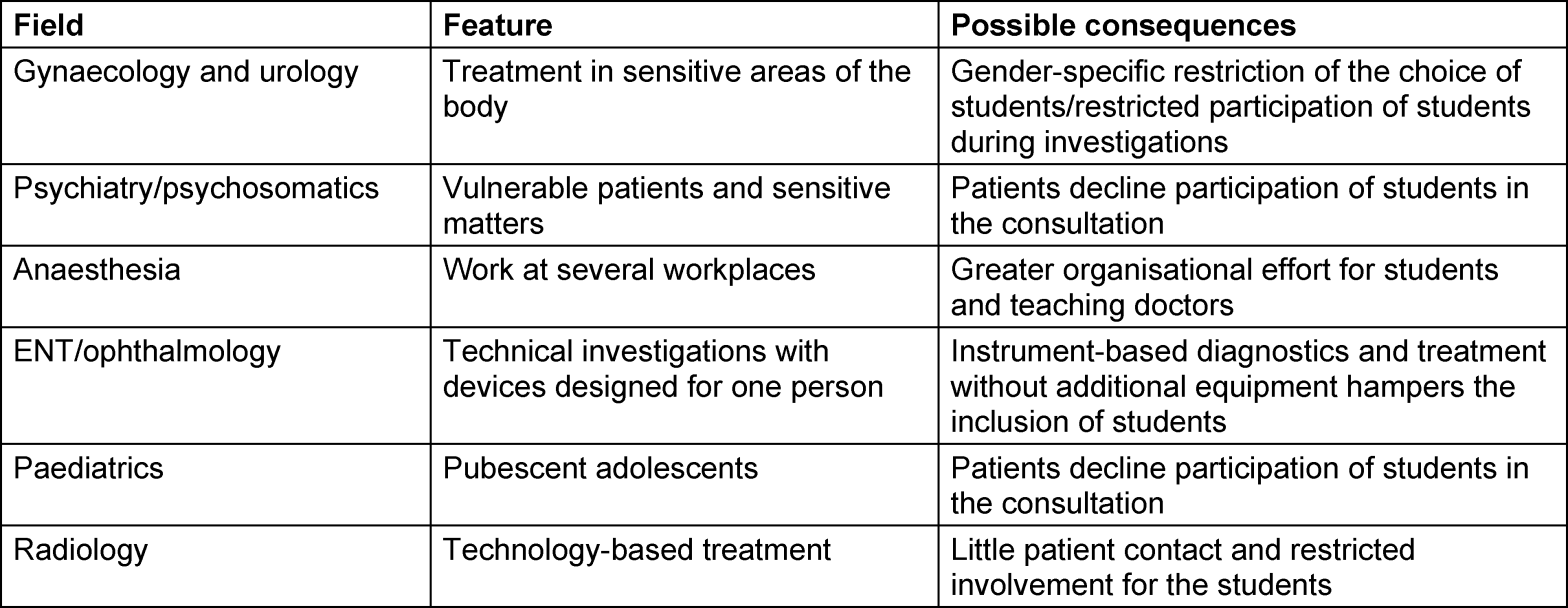
Specialised features and possible consequences
